# Relationships Among Tweets Related to Radiation: Visualization Using Co-Occurring Networks

**DOI:** 10.2196/publichealth.7598

**Published:** 2018-03-15

**Authors:** Ayako Yagahara, Keiri Hanai, Shin Hasegawa, Katsuhiko Ogasawara

**Affiliations:** ^1^ Faculty of Health Sciences Hokkaido University of Science Sapporo Japan; ^2^ Faculty of Health Sciences Hokkaido University Sapporo Japan; ^3^ Sapporo-Kosei General Hospital Sapporo Japan; ^4^ Graduate School of Health Sciences Hokkaido University Sapporo Japan; ^5^ National Institutes for Quantum and Radiological Science and Technology Chiba Japan

**Keywords:** Twitter, social media, public concern, nuclear power plants, morphological analysis, network analysis, radiation

## Abstract

**Background:**

After the Fukushima Daiichi nuclear accident on March 11, 2011, interest in, and fear of, radiation increased among citizens. When such accidents occur, appropriate risk communication must provided by the government. It is therefore necessary to understand the fears of citizens in the days after such accidents.

**Objective:**

This study aimed to identify the progression of people’s concerns, specifically fear, from a study of radiation-related tweets in the days after the Fukushima Daiichi nuclear accident.

**Methods:**

From approximately 1.5 million tweets in Japanese including any of the phrases “radiation” (放射線), “radioactivity” (放射能), and “radioactive substance” (放射性物質) sent March 11-17, 2011, we extracted tweets that expressed fear. We then performed a morphological analysis on the extracted tweets. Citizens’ fears were visualized by creating co-occurrence networks using co-occurrence degrees showing relationship strength. Moreover, we calculated the Jaccard coefficient, which is one of the co-occurrence indices for expressing the strength of the relationship between morphemes when creating networks.

**Results:**

From the visualization of the co-occurrence networks, we found high citizen interest in “nuclear power plant” on March 11 and 12, “health” on March 12 and 13, “medium” on March 13 and 14, and “economy” on March 15. On March 16 and 17, citizens’ interest changed to “lack of goods in the afflicted area.” In each co-occurrence network, trending topics, citizens’ fears, and opinions to the government were extracted.

**Conclusions:**

This study used Twitter to understand changes in the concerns of Japanese citizens during the week after the Fukushima Daiichi nuclear accident, with a focus specifically on citizens’ fears. We found that immediately after the accident, the interest in the accident itself was high, and then interest shifted to concerns affecting life, such as health and economy, as the week progressed. Clarifying citizens’ fears and the dissemination of information through mass media and social media can add to improved risk communication in the future.

## Introduction

On Friday, March 11, 2011, a magnitude 9.0 earthquake occurred 24 km off shore in the Tohoku region in eastern Japan. The tsunami generated by that Great East Japan Earthquake also caused enormous damage. In addition to the loss of human life, serious damage was done to the Fukushima Daiichi Nuclear Power Station. Reactors 1-3, in operation at the time, were automatically shut down, with loss of all electrical power, including emergency power, due to the tsunami. Without power to control the station, there was a reactor core meltdown and hydrogen explosion, releasing the radioactive substances iodine-131 and cesium-137. The Fukushima Daiichi nuclear accident had worldwide repercussions and was classed as a level 7 disaster (severe incident) on the International Nuclear and Radiological Event Scale, similar to that for Chernobyl [1].

Soon after such accidents, public opinions are formed and expressed through various platforms including social networking sites. Twitter is one such platform that allows users to communicate information in tweets up to 140 characters in Japanese language on an individual, specified group, or global basis [2]. Twitter is easy to use because it is available on multiple devices, including mobile phones. People provide and share initial information and real-time situation updates during various crises [3]. The temporal, spatial, and social dynamics of Twitter activity have intrigued many researchers in developing applications to facilitate early event detection and increase situational awareness [2].

In March 2011, there was a large number of Twitter users in Japan. At the time of the earthquake, communications were cut or limited because telephone networks were seriously damaged by the earthquake itself and the subsequent tsunami waters and power loss. Telephone services were also severely affected by heavy use. Twitter was used as a means of communicating information [4]; however, uncertain information, misinformation, and rumors may have contributed to social anxiety and confusion.

Twitter research on the Fukushima Daiichi nuclear accident has focused on the formation of an online community [2], tracking the public mood of the population [5], and sustained interest in accidents [6]. As far we know, however, few studies have revealed specifically what kind of information was spread and what may have incited fear among people dealing with the accident. If an event similar to Fukushima Daiichi nuclear accident were to occur in the future, then understanding changes in people’s concerns and fears early on would be beneficial in providing appropriate governmental responses. To ensure appropriate risk communication [7,8] in such a situation, identifying how people’s interests (especially fears) change over time would be beneficial. Providing appropriate risk management processes in advance may reduce the potential for larger issues emerging.

To assist in appropriate risk communications during future events similar to the Fukushima Daiichi nuclear accident, this study aimed to identify and understand changes over time in people’s concerns, specifically fear, from an examination of radiation-related tweets.

## Methods

### Research Data

The data used in this research consisted of 1,457,230 tweets that included any of the terms “radiation,” “radioactivity,” and “radioactive substance(s)” that were communicated from March 11-17, 2011. Tweets were divided by day.

### Extraction of Relevant Tweets

We used the AWK programming language [9] in this research. AWK is designed to handle text files and is characterized by its powerful capabilities of matching standard expressions. Using AWK, data from each day were processed using the following steps: (1) extraction of only those tweets containing an expression suggesting fear, (2) deletion of user and URL information, and deletion of the spaces before and after tweets, and (3) deletion of identical text.

In focusing on fear-related tweets, 89 expressions were found, including those using Japanese language postpositional particles and parts of speech, as well as expressions using hiragana and katakana Japanese text characters. Table 1 shows examples of the expressions, and the English translation is described with reference to WordNet [10].

Deleting user details, URLs, and extra spaces ensured that only the tweets themselves were extracted. Moreover, since many of the identical texts had spaces before and after said text, these texts would have been considered not exactly identical. These spaces were eliminated.

When deleting duplicate text, identical text was classified as BOTs (ie, abbreviation of “robot”; automatically tweeted items), official retweets (ie, without changes, of another person's tweet), and unofficial retweets (ie, transmission of another person’s tweet with additional comments by sender). As unofficial retweets are considered useful for reading the thoughts of senders, BOT and official retweets were deleted, and only unofficial retweets were used for this study. We classified BOT as a tweet that is linked to “BOT” in the username or profile.

### Morphological Analysis

Morphological analysis is a basic technique used in the field of natural language processing to break down a given text to its morphological elements (ie, morphemes), or the smallest elements in a language that still have meaning. Morphological analysis not only divides given text into morphemes, but it also provides information on the parts of speech and usage, etc, of these morphemes. Morphological analysis is used when one wants to confirm how many times a certain term was used and is indispensable for Japanese text analysis, such as the extraction of keywords.

**Figure 1:**
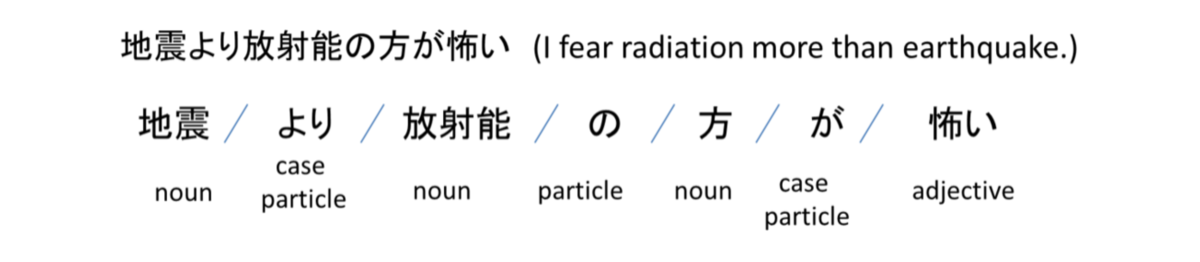
Example of morphological analysis in Japanese.

In this study, we used the morphological analyzer, MeCab (version 0.996) [11], to perform morphological analysis of each tweet that included fear. Figure 1 shows an example of morphological analysis.

**Table 1:** Category of expressions that suggest fear in Japanese (examples).

Japanese	English
恐怖, 怖れる, 恐れる, 畏怖	fear, dread
心配, 不安, 危惧	fear
悪寒	shiver, shake, tremble
絶望	despair, desperation
悲鳴	scream, shriek, screaming, shrieking, screech, screeching
おぞましい, 怖い, 恐い	terrible, dreadful, frightening, awful, fearful, dire, “direful”, dread, dreaded, fearsome, horrendous, horrific
蒼白	pale
案じる	consider, debate, deliberate, moot, turn over
悪夢	nightmare, incubus
やばい, ヤバい	serious, grave, dangerous, grievous, severe, life-threatening
戦慄, 震える	shiver, shudder, thrill, throb
恐ろしい	frightful, awful, terrible, tremendous

**Table 2:** Visualization conditions of co-occurrence networks.

	Conditions
Jaccard index	≥0.1
Number of appearance terms	From 100-150 words
Part of speech	Noun, verb, adjective, adverb, personal name, geographical, unknown part of speech (words not included in the morphological analysis dictionary)
Drawing conditions of network diagram	Strong co-occurrence relationships are thicker lines
	A word with a larger number of occurrences has a larger circle
	Arrangement that labels do not overlap
	Detect subgraph

### Co-Occurrence Rate and Network

Co-occurrence indicates that morphemes A and B were used at the same time. Co-occurrence rate is an index for expressing the strength of the relationship between morphemes. Although other calculation methods exist for ascertaining co-occurrence rates, computations were made in our study using the Jaccard coefficient expressed by the following mathematical formula:

Jaccard coefficient = F
_i_/(F
_1_+F
_2_-F
_i_)

where F_1_ is the frequency of morpheme A’s appearance within all tweets, F_2_ is frequency of morpheme B’s appearance, and F_i_ is the frequency of the simultaneous appearance of morphemes A and B.

A co-occurrence network uses the appearance frequencies and co-occurrence frequencies of each morpheme and is a quantitative depiction of the relationship among morphemes frequently expressed within a text, and morphemes frequently co-occurring with these morphemes. This study provides a graphic depiction of the co-occurrence networks of the relationships between various morphemes appearing in citizens’ tweets. These networks help us ascertain the concerns of the public. This study is limited to only those tweets that include expressions suggesting fear. We used the software, KH Coder [12], for visualizations. Table 2 shows the visualization conditions within KH Coder.

## Results

### Research Data

There were 82,905 tweets (5.7%) excluding BOTs and official retweets. The number of tweets per day and examples of new words that had not appeared before, on each day, are shown in Table 3.

### Co-Occurrence Networks

**Figure 2:**
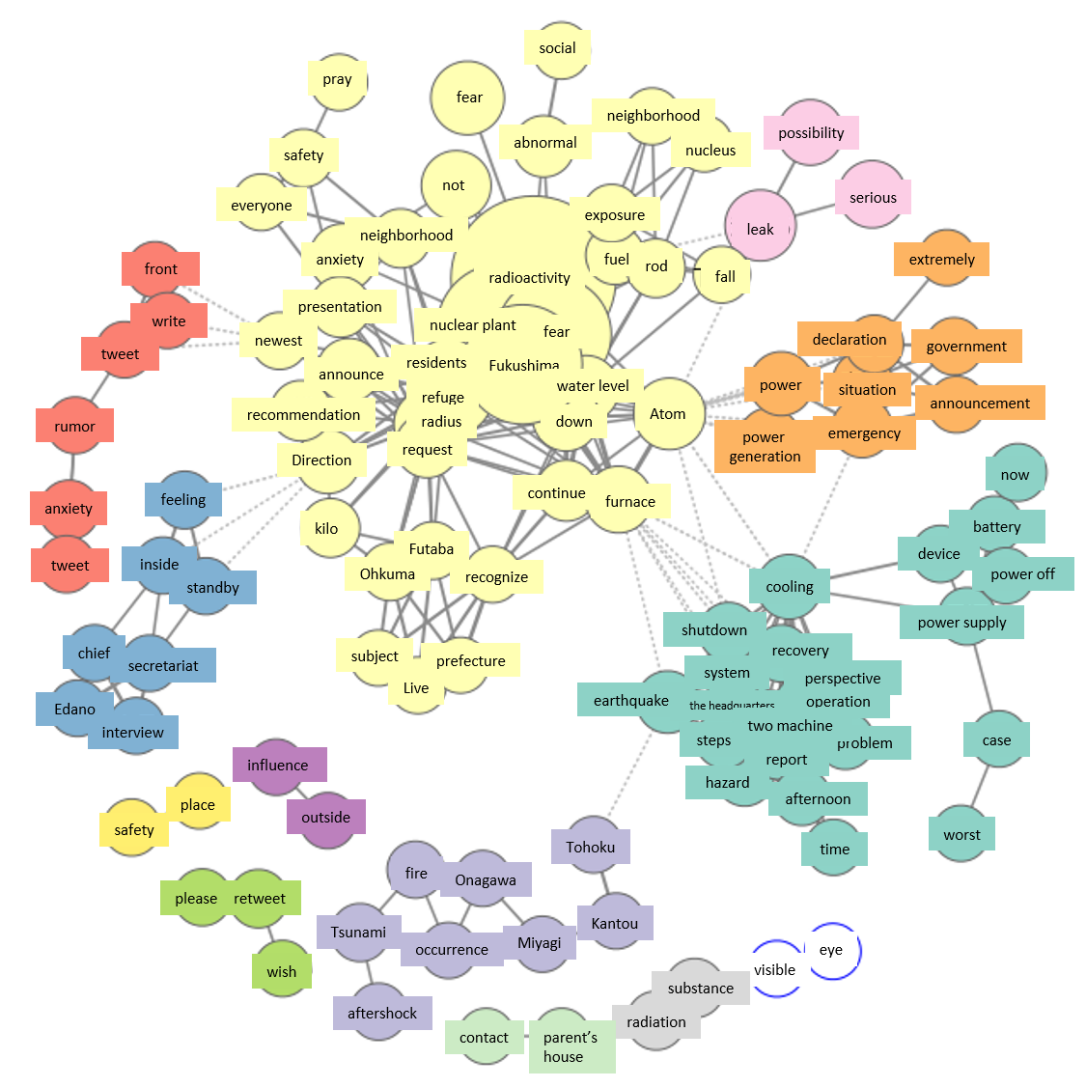
Co-occurrence networks on March 11.

**Figure 3:**
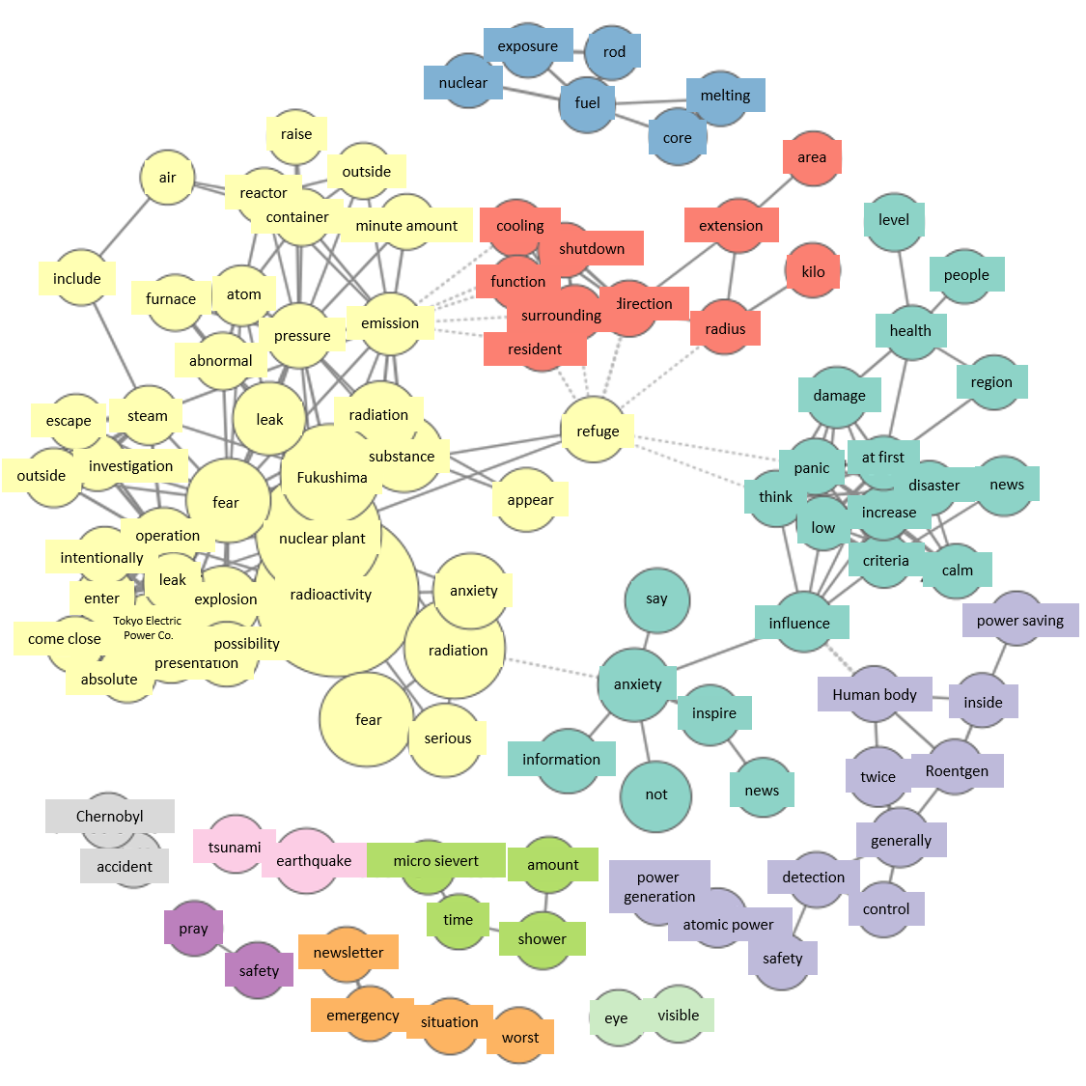
Co-occurrence networks on March 12.

**Figure 4:**
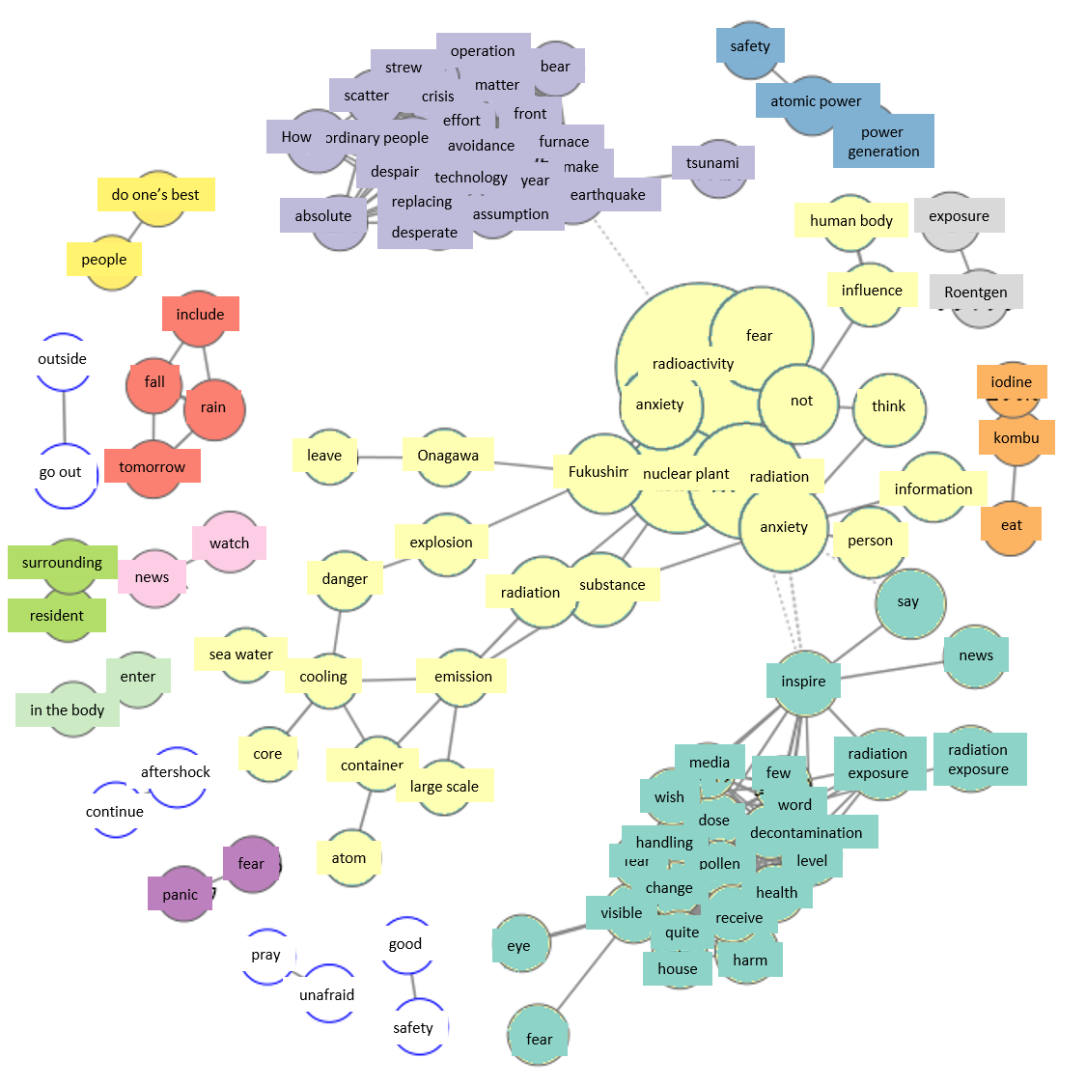
Co-occurrence networks on March 13.

**Figure 5:**
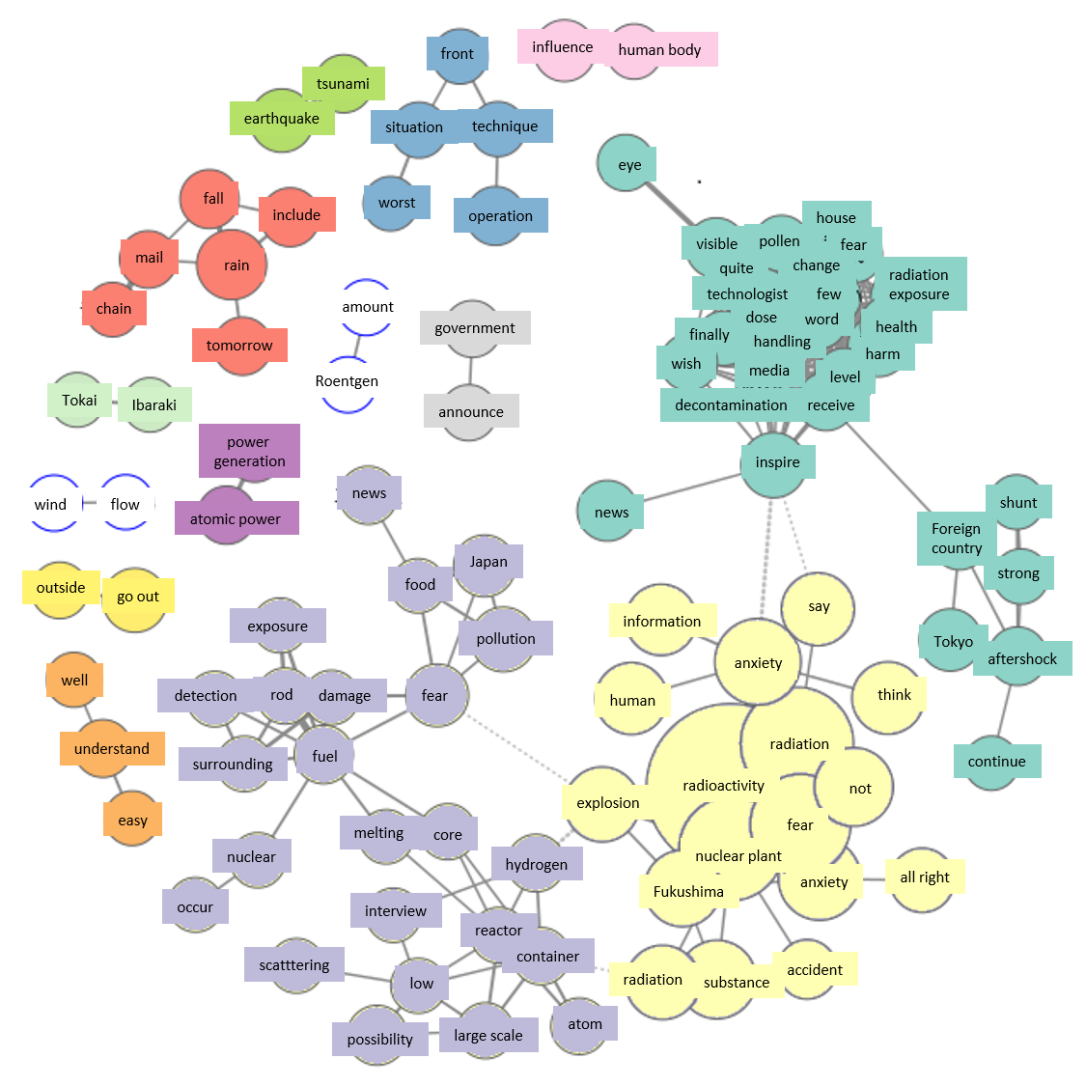
Co-occurrence networks on March 14.

**Figure 6:**
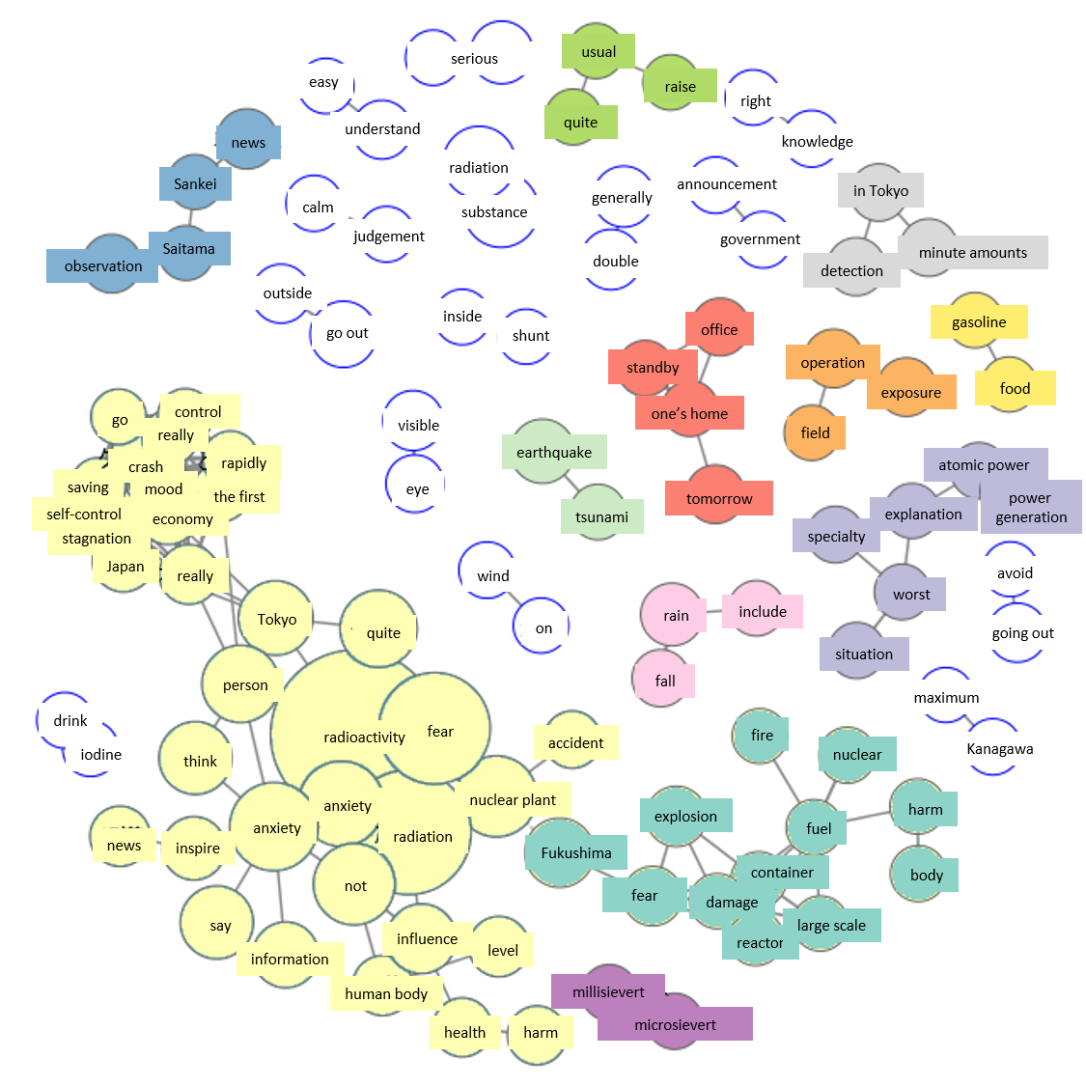
Co-occurrence networks on March 15.

**Figure 7:**
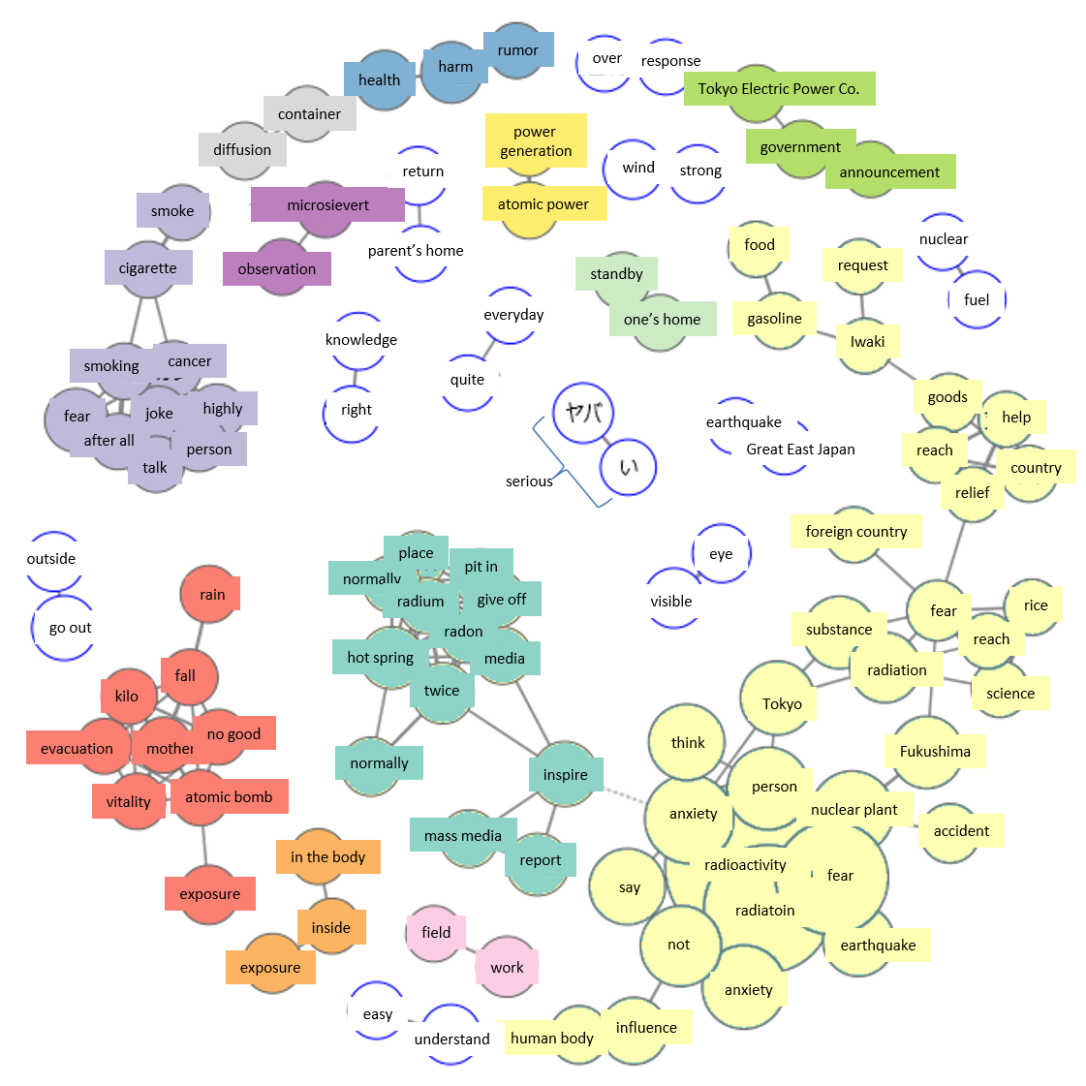
Co-occurrence networks on March 16.

**Figure 8:**
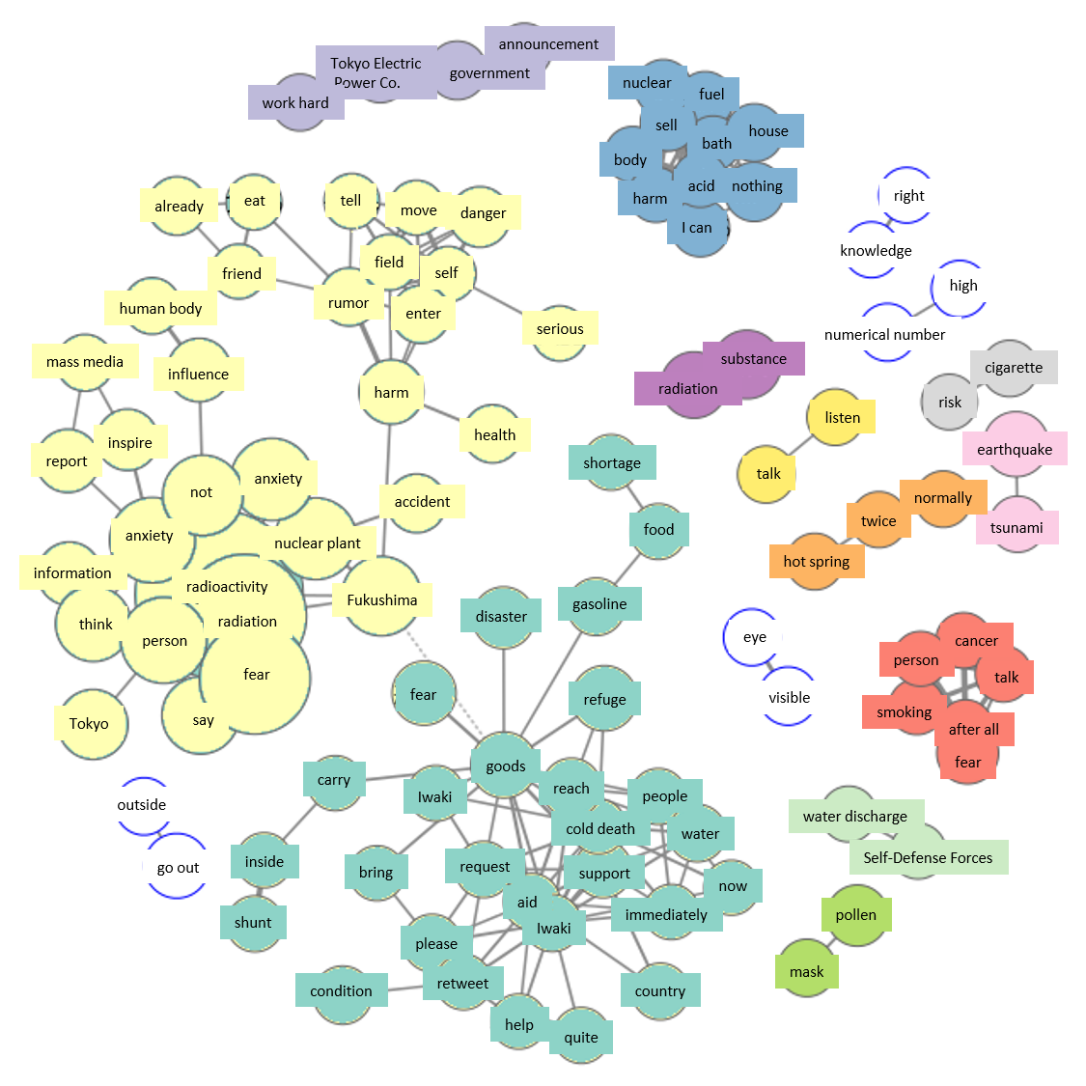
Co-occurrence networks on March 17.

Figures 2-8 show co-occurrence networks for March 11-17, 2011. In the co-occurrence network for March 11, the Fukushima Daiichi Nuclear Power Station group that is highlighted in red shows many Fukushima Daiichi Nuclear Power Station‒related terms and is a characteristically large group formation. Figure 2 shows the existence of tweets that fanned people’s worries.

In the co-occurrence network for March 12, just as for March 11, Fukushima Daiichi Nuclear Power Station again forms a large group. A new group related to health has appeared, centered on the terms “health,” “damage,” “human body,” and “x-rays” (Figure 3).

On March 13, Fukushima Daiichi Nuclear Power Station, which was an extremely large group on March 12, now shows fewer related terms. This suggests the appearance and spread of new areas of concern other than Fukushima Daiichi Nuclear Power Station. The new topic of concern is health, which first appeared on March 12, and mass media (Figure 4).

The co-occurrence network for March 14 again shows citizen concern focused on health and mass media. Also appearing on this day are apprehensions about the impact of radiation spread by winds, as well as radiation contamination of foodstuffs. Other new terms appearing are “Tokyo” and “abroad” (Figure 5).

On March 15, a group of terms appears for material shortages, namely, “gasoline” and “food” (Figure 6).

On March 16, the small group formed the day before on material shortages in Iwaki City (Fukushima Prefecture) has grown to a larger group, with the appearance of new terms, including “Iwaki,” “request” (eg, for assistance),” “material goods,” “support,” “reach” (as in an item arriving at its destination), “government,” “relief assistance,” etc. Also trending this day on Twitter were terms not seen on March 15 or earlier, including “rumor,” “cancer,” “gag” (as in “joke,” but can be negative), and “radium” (Figure 7).

In the co-occurrence network for March 17, the group concerning material shortages in the disaster-hit city, Iwaki, grew in size, while new terms such as “water” and “freeze to death” (literal sense) also appeared. Also, as on the previous day, trending terms were “acid,” “bath,” etc (Figure 8).

Comparing the network diagrams (Figures 2-8) with the new terms of each day (Table 3), the newly appearing groups tended to include newly emerged terms on that day.

#### Friday, March 11

Analysis of the group of tweets concerning “Fukushima Daiichi Nuclear Power Station” appearing in the co-occurrence network March 11 showed that many citizens used Twitter to disseminate reports from the mass media directly after the accident. Thus, media reports had a large impact on citizens, with expansion of the group regarding “Fukushima Daiichi Nuclear Power Station.”

#### Saturday, March 12

Analysis of the group of tweets concerning health in the co-occurrence network for March 12 showed that this was instigated by a statement made by a radiologist on television: “It is difficult to think of any health damage occurring in regions not affected by the evacuation advisory.” Another characteristic of the co-occurrence network were expressions used in the mass media referring to Fukushima Daiichi Nuclear Accident as a “second Chernobyl.” Thus, just as on the previous day, expressions from media reports appeared in the co-occurrence network, meaning that such reports had a major impact on citizens.

**Table 3:** Number of tweets each day and examples of new words appearing each day.

	March 11	March 12	March 13	March 14	March 15	March 16	March 17
Tweets, n	6262	16,430	4879	4922	23,945	16,735	9732
Examples of words	nuclear plant Fukushima fear refuge residents water level earthquake	explosion operation information damage human body Roentgen health	radiation exposure rain technology despair dose media Kombu	pollution Tokyo Japan hydrogen scattering Tokai foreign country	economy observation self-control one’s home gasoline saving food	goods country cancer reach help Iwaki relief	water numerical number shortage self-defense forces acid cold death bath

#### Sunday, March 13

Analysis of the group of tweets concerning health and the media outlined in blue were centered on health-related reports in the media, including discontent with the way mass media presented information, summed up as, “They should not be reporting on whether we have been exposed to radiation, but instead to what extent we have been exposed, and what effects such exposure might have on health.” Also included were statements referring to measures for preventing internal radiation from iodine-131, such as eating seaweeds with trace amounts of iodine. Others worried about the effects of radioactive rainfall. Considering that media report contents had appeared in the co-occurrence networks for March 11 and 12, the group for “Fukushima Daiichi Nuclear Power Station” was smaller on March 13 than on the previous day, with expansion of the group related to health. We believe this reflects the increased media reports about the effects of radiation on the body.

#### Monday, March 14

Analysis of tweets with the words “Tokyo” and “abroad” suggests that foreigners living in Tokyo were evacuating the city for points in Japan west of the capital. One factor is thought to be that reports in foreign media about the nuclear accident were causing people living outside Japan to tell their family members living in Tokyo to evacuate the city. It is thus assumed that the Fukushima Daiichi nuclear accident had become a huge concern in the foreign media and for people outside the country.

#### Tuesday, March 15

Analysis of the new group of tweets concerning “the economy” led to consideration of several factors, including reports in the Sankei Shimbun newspaper of “radiation reaching 20 times normal levels in Saitama and 40 times normal levels in Tokyo,” and the fact that a famous entrepreneur, Takafumi Horie, had tweeted his opinions regarding the effects of radioactive substances on the Japanese economy. Tweet analysis also showed that citizens were agreeing with the thoughts expressed by famous people on Twitter. This suggests that even after such an accident, celebrities had a major impact on ordinary Japanese citizens. Analysis of the group of tweets concerning material shortages showed that, due to gasoline and food shortages, many of the citizens of Iwaki City were unable to evacuate and were forced to stay in that city.

#### Wednesday, March 16

Analysis of the material shortages group of tweets, which had expanded from the previous day, showed that accident areas were impacted by rumors that the news media and materials transport companies were hesitating to enter accident areas. Reports in the media about material shortages first appeared March 16. In addition, the fact that the material shortages group was already appearing in the March 15 co-occurrence network suggests that trending issues on Twitter can appear in a co-occurrence network without the influence of media reports. Newly appearing terms on this day, such as “cancer,” “gag,” and “radium,” were the result of numerous people in the surrounding areas sending unofficial retweets.

#### Thursday, March 17

The increased size of the material shortages group in accident areas on this day compared to the previous day was due to the increase of terms related to such material shortages. It seems to have occurred as the result of media reports of Fukushima Prefecture’s official request to the national government for relief. People surmised, therefore, that this request reflected the lack of goods in accident areas. This group was the result of unofficial retweets, just as with the terms “cancer” and “gag” on March 16.

### Sources of Citizen Concern

We analyzed changes in citizens’ concerns in the visualization of the co-occurrence networks, finding the following changes in areas of concern: March 11 and 12, “nuclear power plants”; March 13 and 14, “health,” “media”; March 15, “economy”; March 16 and 17: “material shortages in accident-stricken areas.” Next, we tried to understand how such concerns grew in importance by identifying the mechanisms behind the occurrence of these concerns.

Concerns about “power plant,” “health,” and “mass media” are thought to be due to the fact that citizens took information from mass media sources and then transmitted them on Twitter with their own opinion added. Table 3 shows a timeline of power plant accident‒related words.

Groups concerned with “power plant” appearing on co-occurrence networks were the result of daily, detailed reports in the media following the accident. Health-related groups appeared from March 12 on. Although an evacuation advisory was issued on March 11 for a 3 km area surrounding the plant, this is deemed to have been too small for people to have become concerned about their health. On March 12, however, the media reported that the evacuation zone had been extended considerably to 20 km, and this is thought to have stirred up citizens’ concerns for their health. Further, starting March 13, the media fanned people’s fears by repeatedly stressing that the accident was “at the same level as Three Mile Island,” or that radiation levels had increased “by several 10s or even 100s of times above normal,” resulting in the increase in health-related groups in the co-occurrence networks. People are assumed to have turned their attention to the mass media because it was producing reports that incited people’s anxieties.

The term “economy” appeared in the March 15 co-occurrence network. Among the days studied, March 15 was the only day when special concern for the economy became a trending topic. Analysis of related tweets showed that Takafumi Horie had used Twitter to communicate his opinions. His tweets reverberated among citizens:

The worst thing that could happen would be for people to leave Tokyo and slow down the economy, or cause the economy to stagnate due to a mood of self-restraint. Frugality and self-restraint will certainly cause the Japanese economy to crash. And that is more fearful than radioactivity.

People began to worry about the economy, which was manifested in such tweets as:

I’ll keep working on what I can do tomorrow.

Tokyo and Saitama are far from Fukushima. Rather than worrying about radiation, pour your whole heart and soul into the rebuilding of the economy! Support the nation’s infrastructure, keep lots of money in circulation, and send lots of assistance money to accident-stricken areas. That is what we can do now. That is our mission.

It is thus clear that citizens’ concerns about the economy did not result from mass media reports, but instead from information communicated on social media sites like Twitter.

As for the co-occurrence network groups concerning material shortages in the accident zone city, Iwaki, there was an increase over the days in number of related terms used, even though they were all related to material shortages. This was a major characteristic of the co-occurrence networks. On March 15, the concern was about “gasoline” and “food”; on March 16, the terms were “Iwaki,” “gasoline,” “food,” “materials”; on March 17, these were “Iwaki,” “gasoline,” “food,” “materials,” “water,” and “shortage.” Analysis of material shortages‒related tweet groups showed that people in accident-stricken areas made requests for assistance:

Please, somehow understand that, even if we are instructed to evacuate, almost none of the citizens here have the means to evacuate! There is no gasoline left in this city. We do not have the water we need to wash away the radioactive substances

official retweet request; from Iwaki City

We ask for help. The tsunami damage along the coast in Iwaki City, Fukushima Prefecture, as well as in Ibaraki Prefecture, which are parts that haven’t received much coverage, has left us hopeless. The media won’t report our situation because they are afraid of radiation. We don’t have any food or gasoline.

After receiving these tweets from accident victims reporting on the accident situation, people in other areas began to send tweets requesting people to restrict their use of materials: “Harmful rumors are circulating about Iwaki City. It seems that excessive fears of radiation have caused a halt to transports into the city. Please conserve gasoline!” From the above, it is clear that the concern about “material shortages in accident-stricken areas” was not fostered by the mass media, but rather through the transmission of information on Twitter. On March 15, there were no reports in the media about material shortages being a serious problem. Only with the announcement on March 16 by Yukio Edano, then Chief Cabinet Secretary, did the media first report such shortages. This is thought to have stemmed from the harmful rumors circulating about Iwaki City, such that the news media and materials transport companies feared radiation exposure if they entered that city. However, that such shortages appeared on the co-occurrence network for March 15 suggests the possibility that people learned of the worries of Iwaki residents via Twitter more quickly than they did from media reports.

## Discussion

### Principal Findings

In this study, we analyzed trends in citizens’ focus on Twitter after the Fukushima Daiichi nuclear accident. A better understanding of citizens’ fears after such an accident can assist in improved risk communication and government response in similar situations in the future. Based on the results of this study, we will attempt to estimate the number of tweets before the accident and analyze them in order to compare the data and clarify the changes in citizen interest before and after the accident.

### Limitations

One point to be improved in this study is that of optimization of extraction conditions. One of Japanese expressions for fear, “恐れ,” has two meanings: one is “fear” and the other is “dread.” By using “恐れ” as the extraction term, it is possible that tweets with “fear” meaning “possibility” were mixed with those about “fear” meaning “dread.” For example, many Fukushima Daiichi nuclear accident‒related media reports included the use of “fear” with its meaning of “possibility,” as in, for example, “there is fear of radiation leakage.” People who encountered such reports may have therefore been influenced in the tweets they then sent. Thus, unnecessary terms that did not express people’s fears (anxieties, dread) might have appeared in this study’s co-occurrence networks. For future research, it will be necessary to optimize term extraction conditions.

### Conclusions

This study used Twitter to understand changes in Japanese citizens’ concerns in the week following the Fukushima Daiichi nuclear accident, with a focus specifically on citizens’ fears. Immediately after the accident, the interest in the accident itself was high and then interest shifted to concerns about life, such as health and the economy throughout the following week. Understanding citizens’ fears and the dissemination of information through mass media and social media can add to improved risk communication in the future.
